# Trusted Operations on Sensor Data †

**DOI:** 10.3390/s18051364

**Published:** 2018-04-27

**Authors:** Hassaan Janjua, Wouter Joosen, Sam Michiels, Danny Hughes

**Affiliations:** imec-DistriNet-KU Leuven, Celestijnenlaan 200A, B-3001 Heverlee, Belgium; Wouter.Joosen@cs.kuleuven.be (W.J.); sam.michiels@cs.kuleuven.be (S.M.); Danny.Hughes@cs.kuleuven.be (D.H.)

**Keywords:** Trusted Execution Environment (TEE), Rich Execution Environment (REE), TrustZone, secure world, normal world, trusted application, authenticity, fidelity

## Abstract

The widespread use of mobile devices has allowed the development of participatory sensing systems that capture various types of data using the existing or external sensors attached to mobile devices. Gathering data from such anonymous sources requires a mechanism to establish the integrity of sensor readings. In many cases, sensor data need to be preprocessed on the device itself before being uploaded to the target server while ensuring the chain of trust from capture to the delivery of the data. This can be achieved by a framework that provides a means to implement arbitrary operations to be performed on trusted sensor data, while guaranteeing the security and integrity of the data. This paper presents the design and implementation of a framework that allows the capture of trusted sensor data from both external and internal sensors on a mobile phone along with the development of trusted operations on sensor data while providing a mechanism for performing predefined operations on the data such that the chain of trust is maintained. The evaluation shows that the proposed system ensures the security and integrity of sensor data with minimal performance overhead.

## 1. Introduction

The idea of participatory sensing using mobile phones has been around for more than a decade [1]. The concept was inspired by the huge number of mobile devices that were already present at that time. Now after nearly 11 years, the situation has become even more favorable for participatory sensing with more than four billion mobile phones and more than two billion smartphones being used across the world [2]. The huge number of readily available mobile sensing and computing devices provides enormous opportunities to deploy widespread low-cost sensing infrastructure for collecting data from participants who would voluntarily provide sensor data and feedback in exchange for services or rewards [3]. Moreover, these mobile phones can act as a gateway to relay data collected by sensors that are not part of the mobile phone itself, but connected to it via a short-range connection.

Participatory sensing provides many advantages over dedicated sensing infrastructure. Since mobile phones and their communication infrastructure are already present, the marginal extra cost of sensor infrastructure deployment is only limited to the development of the mobile application that will be used to collect and relay the sensor data to a central server. External sensors can also be connected easily to a mobile phone and still utilize the mobile phone communication infrastructure to relay the sensor data. Adding external sensors to a data acquisition framework enables a wide range of possible applications ranging from personal health data collection to environmental monitoring. Since participatory sensing could potentially assimilate anyone using a mobile phone, it has the capacity to support much larger systems. Participatory sensing also creates the opportunity to use people as qualitative sensors that collect user feedback and opinions where subjective data make more sense than objective values; a practical example of such a system is the Google Local Guide program [3].

Participatory sensor data collection often require a mechanism to ensure the integrity of the sensor data; as it is not feasible to trust all participants. In many cases, participants can be motivated to provide false or repetitive data to gain benefits or to simply disrupt the system with malicious intent. Another real-world example is the Joyn [4] community. The business model of Joyn revolves around participatory sensing of data by volunteers in exchange for rewards such as discount coupons, free coffee, mobile top up, etc. The more data volunteers collect, the more rewards they could achieve. This provides a clear motivation to report false data in order to achieve rewards. Fabricating false sensor data such as image data is quite straightforward by using off-the-shelf tools such as Photoshop [5] and Pixelgarde [6]. Furthermore, a malicious user may use malware to compromise a smart phone’s software stack to inject false data into the participatory sensing application.

The question of the trustworthiness of participatory data was first raised in 2009 by Dua et al. [7]. Since then, many approaches for adding trust to the sensor data have been introduced [8,9,10,11,12,13,14]. These approaches only address the problem of the trustworthiness of raw sensor data, but they fail to provide a practical solution because raw sensor data are rarely used in real-world scenarios. In many cases, these data need to be preprocessed on the mobile device itself in order to make it usable. The authors of [13] addressed the need for preprocessing sensor data while maintaining trust. However, this approach only covers image and voice data and is limited to non-rooted Android systems.

Connecting external sensors to a mobile phone introduces more security requirements. As opposed to mobile phones’ on-board sensors, external sensors need to prove their identity to the mobile phone and establish a trusted communication channel with the mobile phone. Additionally, when sensitive sensors such as medical sensors are attached as external sensors to the mobile phone, the security requirements become much more strict. Beside ensuring the integrity of the data, the secrecy of the data is often a key security requirement for medical sensors, as the unintended disclosure of these data to a malware that is sniffing the communication channel would violate the participants privacy.

In our approach, the assurance of secrecy and integrity is provided by placing the root of trust in the Trusted Execution Environment (TEE) in the mobile phone. TEE ensures security by exposing only a limited interface to the outside world and, as a result, reduces the attack surface. This reduced attack surface is relatively easy to maintain and chances of introducing vulnerabilities that could potentially compromise the security of the system are significantly reduced. On the other hand, we have a contradictory requirement of performing operations on sensor data inside the TEE. Our solution to this problem is to introduce a generic framework that provides the common functions for verifying and signing the data and to provide a unified mechanism to implement trusted operations; as a result, this framework can be rigorously tested and verified independently. This approach is useful because all signing and the verification code are implemented only once for all types of sensor data operations, thus reducing the attack surface, as well as the amount of code to be verified, as compared to implementing the signing and verification for each sensor. Our framework is capable of performing arbitrary operations on sensor data repeatedly while keeping track of the performed operations, maintaining the chain of trust. While TEE ensures the security of data within the mobile phone, we assume that an external sensor device has its own security mechanism, and we can rely on the sensor node’s ability to protect sensor data and the cryptographic keys from malicious access.

The key contributions of our paper are (1) a practical framework that provides the secrecy and integrity of sensor data and (2) allows arbitrary operations to be performed on the data while maintaining secrecy and integrity after the operations. (3) Our framework also supports binding two or more sensor data sources together in a trustworthy manner. (4) We are first to identify a set of security considerations for mobile phone external sensors that are necessary to provide secrecy and integrity guarantees for the data that are being generated on the external sensor in a unique setting, where the adversary is able to compromise the security of the operating system. (5) We also propose a protocol for communication between the TEE and external sensor that satisfies the security needs of this system. The framework presented in this paper provides secrecy and integrity guarantees by utilizes two basic security operations simultaneously, seal and attestation, as presented in [9]. For the sake of simplicity, we refer to these two operations by the term seal and the corresponding inverse operation by unseal.

The remainder of the paper is organized as follows. Section 2 describes the background on the trusted execution environment, ARM TrustZone technology and the available secure world operating systems. In Section 3, we provide related work in trusted sensors data and approaches for the maintenance of integrity in the modified sensor data. Section 4 describes a real-world scenario with use cases that implement our solution and chart out the requirements for the system. In Section 5, we define the threat model that our system addresses and define the extent of the capabilities of an adversary. Then, we provide the design of the framework and the protocol for external sensor communication in Section 6, followed by the performance and security evaluation of our framework in Section 7. Finally, in Section 8, we provide our conclusion.

## 2. Background

A Trusted Execution Environment (TEE) is an isolated secure environment that provides processing, memory and storage capabilities that are protected from the operating system, which is termed as the Rich Execution Environment (REE). TEE requires hardware support to provide protection from the REE. Many embedded hardware solutions are available for implementing TEE from various vendors. The hardware solution provided by ARM is TrustZone [15]; Intel provides Software Guard Extensions (SGX) [16]; AMD provides the AMD Secure Processor [17]. A TEE operating system manages the security hardware and provides a means to communicate between the TEE and REE. Many TEE operating systems are available as open source, as well as proprietary software such as OP-TEE by Linaro [18], Trustonic [19], Qualcomm QSEE, T6 by TrustKernel [20] and SecuriTEE by Hansol Secure [21]. GlobalPlatform addresses this heterogeneity in TEE by providing a set of standardized technical specifications, which facilitates development on various hardware platforms.

### 2.1. ARM TrustZone

**Figure 1:**
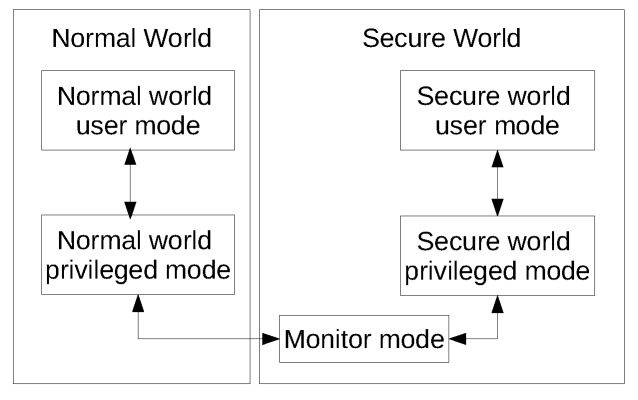
ARM TrustZone secure and normal worlds.

ARM TrustZone is a hardware-based execution environment isolation technology. TrustZone uses the concept of secure and non-secure worlds that are hardware separated, with non-secure software blocked from accessing secure resources directly. This separation of secure and non-secure worlds extends beyond the processor to encompass memory, software, bus transactions, interrupts and peripherals within an SoC. As illustrated in Figure 1, both secure and non-secure worlds have their own user mode and privileged mode, while the switching between the secure and non-secure worlds is done via a special mode called the monitor mode. The approach to security in TrustZone is that the rich operating system runs in the non-secure world, while a small and secure operating system runs in the secure world. The solution proposed in this paper relies on hardware-based execution environment isolation technology. This dependence does not hinder the widespread deployment of our proposed solution because the said hardware-based isolation technology is already present in the majority of smart phones currently available on the market.

### 2.2. GlobalPlatform Trusted Execution Environment

GlobalPlatform is an international cross-industry technical standardization body, and it facilitates secure deployment and management of embedded applications. GlobalPlatform has standardized the trusted execution environment, which is a secure area in the main processor in an embedded device. TEE provides an isolated environment where sensitive data can be stored and processed. This isolated trusted environment is capable of providing end-to-end security by only allowing trusted and authenticated code to be executed in this environment. The TEE API specifications are independent of the underlying hardware implementation and provide a generalized set of APIs that can be used to implement hardware independent trusted applications. The framework presented in this paper utilizes the TEE internal APIs to implement a trusted application and TEE client APIs to communicate between the secure and normal worlds [22].

### 2.3. Secure Operating System

A secure operating system runs in the secure world in parallel with the normal world operating system. It normally has a small footprint and a well-defined communication mechanism with the normal world operating system. The smaller code base and limited communication interface reduce the attack surface of the system, making it difficult to compromise the security of the system. GlobalPlatform has standardized TEE by providing API specifications for secure OS functionality. We have designed our framework to work around the GlobalPlatform’s TEE APIs so that the implementation is independent of any specific operating system.

**Figure 2:**
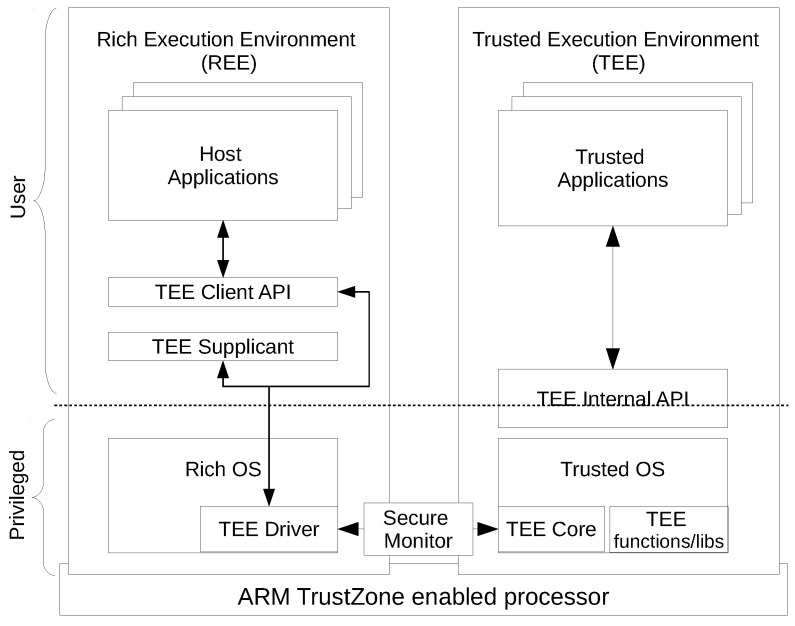
OP-TEE.

Many secure world operating systems implement the GlobalPlatform TEE specifications. OP-TEE is an open source secure operating system that complies with the GlobalPlatform TEE specifications. OP-TEE is ported to a number of TrustZone-enabled ARM-based platforms. A graphical representation of various components of OP-TEE is presented in Figure 2. In addition to the trusted operating system, OP-TEE also has normal world client-side APIs and OP-TEE Linux drives. OP-TEE provides runtime libraries and TEE internal APIs to support execution of Trusted Applications (TA) in secure world user mode. The normal world client side of OP-TEE provides TEE client APIs to support communication with TA running in the secure world. The framework presented in this paper resides in the secure world, where it is provided as a library that compiles with a trusted application. The framework implements TEE life-cycle events to provide an interface for the normal world.

## 3. Related Work

The need to authenticate sensor data was quickly realized following the introduction of participatory sensing. The issue of attesting sensor data has been addressed using many approaches. One such approach is location proofs, which use Wi-Fi access points to generate the proof of position [23]. The relatively short range of the wireless infrastructure is used as a guarantee of location for the physical device. This technique is only possible due to the nature of location data, but it cannot be generalized to other sensors. Furthermore, this is not a strong proof of location against a capable adversary.

A more generalized approach is the use of a dedicated Trusted Platform Module (TPM) to collect and authenticate sensor data. As shown in [7,8], an external TPM can be used to establish trust for the sensor data integrity. The approach proposes that an external TPM is used to sign the raw sensor data. Due to the dependence on the external hardware requirement, this solution is difficult to deploy at a large scale. In contrast, the solution presented in this paper utilizes the TEE already present on the mobile phone as TPM and makes the system much more scalable.

Following the introduction of hardware-based isolated environments such as ARM TrustZone [15] and Intel SGX/TXT [16], new approaches were proposed that utilized these hardware-based isolated environments to add fidelity certificates to sensor data. The work done in [9] uses TrustZone to ensure the trustworthiness of the mobile sensor data. It introduces software abstractions for establishing the trust and confidentiality of the sensor data. The framework conceived of in this paper ensures the trustworthiness of sensor data, but does not address the usage of these data. Although the issue of operations on trusted sensor data is discussed in the context of providing differential privacy, the framework lacks support for generic operations on sensor data while maintaining trustworthiness. The proposed framework is an extension to this work, in particular where we propose a comprehensive framework to not only acquire trusted sensor data, but also provide a mechanism to ensure trust after performing operations on that data.

A practical implementation of trusted operations on images is provided in [10]. The framework uses the License verification library provided by Google [24] to validate the authenticity of the application itself and implements Android keystore features provided on top of ARM TrustZone to store keys and perform signing operations. Although this technique utilizes the ARM TrustZone, it fails to exploit the full potential of the trusted execution environment, and as a consequence, it is vulnerable to attackers that have gained root privileges on the system. Malware installed on the system can hook the image-capturing APIs and feed modified images to the system. In order to protect the system from such a threat model, we need to move the origin of the data inside the TEE.

Executing arbitrary operations inside the TEE adds the additional cost of certifying more code for the TEE and increases the attack surface for a potential adversary. We believe, however, that including this code in TEE has more benefits than the risk it adds. As shown in [11], it is feasible to implement image operations inside the secure world of ARM TrustZone, but it utilizes this ARM TrustZone at the cloud server in order to provide an isolated environment to perform image operations. Our work is the inverse of that: we propose the usage of TEE at the mobile client end to provide trust guarantees.

The work presented in [13] addresses the same problem for images and sound data as in the current paper, but it implements a different approach and a lenient threat model, which assumes that the sensor data cannot be modified at the device driver level. The approach taken by [13] is that it allows arbitrary modifications on the sensor data by any means and later compares the modified data with the original and generates a signature after checking for meaning-preserving modifications. The weak link here is the mechanism of capturing full fidelity sensor data that relies on the Android security system. If we allow a more realistic threat model where the normal world operating system is compromisable, we cannot rely on the fidelity of the sensor data that were originally assumed to be trusted. Furthermore, [13] only addresses the operations performed on image and sound data, but lacks generalized operations for all types of data. Although this technique proposes the utilization of TrustZone in order to perform image operations, it does this to provide protection from malicious access rather than providing trust; whereas the current paper proposes a scheme that provides trust guarantees even when the normal world operating system like Android has been completely compromised. This is achieved by maintaining the root of trust in the TEE.

Plug-n-trust [25] presents a solution to the confidentiality and integrity of medical sensing data along with a framework to perform data processing on a mobile phone. The approach taken by the authors is to provide security guarantees via a smart card that is plugged into the microSD slot of a mobile phone. The smart card serves as a tiny trusted third-party to provide protection from any malicious access from the mobile phone. The sensor data are transmitted to the mobile phone using a short-range communication channel, and all processing on the data is done in the smart card. The solution proposed in this paper is similar to Plug-n-trust; however, instead of placing the root of trust in an external smart card, we propose utilizing the TEE present on the mobile phone. Additionally, as opposed to Plug-n-trust, our system does not require a real-time clock to be present on the peripheral; instead, we establish a proof of freshness to correlate the time of reading of the sensor data.

Table 1 summarizes the capabilities of existing frameworks for providing sensor data integrity, secrecy, support for arbitrary operations on sensor data, the root of trust placement, resilience to a compromised OS and external hardware requirements. As seen in Table 1, the current paper provides all the required features without specialized external hardware, making it scalable while fulfilling all requirements.

## 4. Scenario

This work is motivated by a real-world industrial project for Mobile Sensing seRvices for developing Geospatial IoT Applications (SeRGIo) [26]. SeRGIo aims to transform the role of a reliable mobile workforce (e.g., courier workers) into an intelligent human sensor network supported by an enabling mobile sensing framework for better understanding of citizens and cities.

Many service providers require their workers to move around a city on a daily basis, for example courier workers, door to door salesmen, taxi drivers, etc. There is a huge potential of using this workforce in the field to collect a vast amount of data, as well as to execute small activities such as taking pictures and collecting noise samples from around the city. Such service providers have the potential to enter the market as a provider of citizen and city data for a variety of stakeholders.

These services can task their workforce to collect these data using sensors available on smart phones, or dedicated sensor devices that connect to a smart phone to relay the sensor data. These data will become much more valuable if guarantees for security, authenticity and trustworthiness are provided to the customers. Furthermore, sometimes the sensor data are sensitive, and only authorized access should be allowed; this includes protection from any malware that might be present on the smart phone itself. In order to realize these objectives, we identified the following requirements that need to be fulfilled.

1. Make sure that sensor data are indeed taken from the sensor at the specified time (trust).
2. Keep track of the operations performed on the sensor data and provide guarantees that only the said operations have been performed on the data.
3. Merge trusted sensor data from multiple sensors to provide derived data along with its own trust signature.
4. If required by the application, provide sensor data confidentiality so that the data are only accessible by the trusted components.

In order to provide the proof of fidelity for external sensors, we need to make sure that the data collected by the sensor were collected within a reasonable time boundary. This brings us to the following requirements specific to external sensors.

5. Proof of freshness: The data were collected within a designated time boundary.

Since we utilize TEE as the platform to provide our solution, this choice imposes other requirements of small footprint and smaller attack surface. Additionally, we do not want too much degradation in performance by providing trust guarantees. This leads us to the following requirements.

6. Make sure that the overall footprint of the security code is minimal.
7. Make sure that the trust guarantees do not introduce unacceptable performance overhead.

We fulfill these requirements by providing a framework to help implement capturing of the sensor data, calculate fidelity signatures, encrypt/decrypt the sensor data and implement operations on the data while maintaining trust and confidentiality guarantees. This framework will abstract over the implementation details of calculating and verifying the trust signatures and encryption and decryption of the data from the developer of these operations. We also provide a secure protocol that will be used to communicate sensor data between the external sensor and the mobile phone with built-in capabilities to provide the freshness guarantee.

## 5. Threat Model

We present the security claims of our framework with the help of multiple attacker models, each having increasing control over the device and ability to intercept communication between the sensor and the smart phone. The goal of the adversary here is to either inject fabricated sensor data into the system without being detected or to gain access to unencrypted sensor data. On the other hand, our goal is to detect the fabricated data and to prevent the attacker from gaining access to confidential data. Our system relies on the root of trust provided by the security of the external sensor and the trusted execution environment present on the smart phone. We limit our adversary model by assuming that it is impossible for the adversary to bypass the secure execution capability provided by the TEE and the external sensor, nor is the attacker able to access secret keys stored in the TEE and the external sensor node. This means that the system can be compromised if the security of TEE, or the external sensor node is breached, or the secret key is stolen by means of a side channel attack. Side channel attack is an open problem and is a topic of ongoing research with exploits and solutions emerging continuously [27,28].

Initialization of asymmetric cryptographic keys and distribution of the public keys is an important step in the trust establishment. The trusted operations framework involves four separate entities in trust establishment: the trusted application that resides in the TEE of a mobile phone, the REE application running in the normal world operation system, the verification server, which is an independent server accessible to the TA via REE application, and the external sensor node. We assume that the TA, the sensor node and a verification server have their own pairs of asymmetric keys. The public key of the verification server has been transported to both the TA and the sensor node; the public keys of the sensor node and the TA have been transported to the verification server; and the public key of the TA has been transported to the data consumer. It is possible to include the distribution of these keys in the protocol by utilizing a third party certification authority, but we make these assumptions to simplify the explanation of the key distribution mechanism among the TA and the external one in our framework. Such a distribution of public keys will allow identity verification of the sensor node and the TA to each other and facilitate the authentication of the sealed data by the data consumer. We further assume that the private key of the TA is stored in the secure storage of the TEE, while the private key of sensor node is also secure and cannot be leaked. The root of trust is placed on the security of the external sensor and the TEE in the mobile phone, and tampering with or stealing the key from the TEE or the external sensor is out of scope of this paper.

### 5.1. Controlled Devices

We first analyze our system when an adversary is trying to falsify sensor data on a controlled device such as a device that is provided to the service provider employees, and it is closely monitored. In this case, the adversary only has control over the user mode applications, since any attempt to root an Android device or to jailbreak an iOS device will be detected by the administrator. In this case, the adversary has no control over the sensors and cannot introduce fabricated sensor data at the time of data acquisition. However, the adversary can play a man-in-the-middle attack by placing itself between the application and the back-end server, but in this case, the server will be able to detect falsified data based on the signatures.

### 5.2. Bring Your Own Device

It is easy to acquire root privileges in Android or jailbreak an iOS device either using malware or due to the actions of the user. We assume that in this case, the adversary has full control over the normal world operating system, but does not have access to the TEE. In this scenario, the adversary again can only tamper with the sensor data, but does not have access to the secret keys and, thus, is unable to bypass the attestation mechanism.

### 5.3. External Sensors

Adding external sensors to mobile phones introduces new attack surfaces that need to be taken care of. Similar to on-board sensors, (1) we need to make sure that the sensor data indeed originated from the designated sensor (trust); and (2) that the sensor data are only visible to the intended parties, and lastly; (3) we need to make sure that the data are not older than a predefined time threshold (proof of freshness). A straightforward approach to provide the security of the external sensor data is to use a secure communication channel between the sensor and the mobile phone such as Bluetooth Low Energy (BLE). Although BLE security can provide a secure communication channel between the mobile phone and the external sensor, this security is not enough, because an adversary that has already compromised the Rich Execution Environment (REE) can very easily obtain access to the unencrypted data. We rely on the ability of the external sensor to protect its private key and to prevent the execution of malicious code on the sensor node. Furthermore, we assume that the sensor node is placed within reasonable proximity to the mobile phone. This assumption becomes relevant when the sensor data from the external sensor are to be combined with the location of the mobile phone. A distance bounding protocol such as Brands and Chaum [29] can be used in conjunction with our approach to establish a proximity proof with the mobile phone; however, we will not discuss distance bounding in the context of this paper and leave it as future work.

## 6. Design

The trusted operations framework has three components: (1) the trusted operations library that is implemented in the trusted execution environment of a mobile phone; (2) a sensor data life-cycle is defined, such that the security guarantees are maintained throughout the data acquisition to consumption; (3) the external sensor protocol that defines the communication mechanism between the TEE in the mobile phone and external sensor node such that the similar trust guarantees are maintained with external sensor data as are provided for the on-board sensors of the mobile phone.

### 6.1. Trusted Operations Library

**Figure 3:**
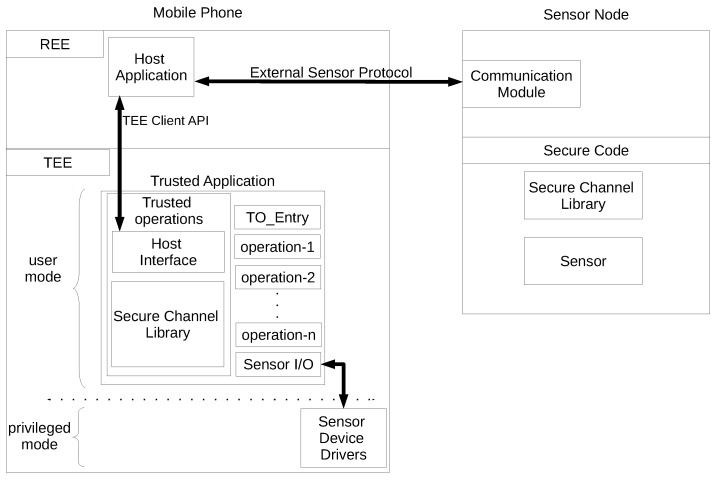
Trusted operations library.

The trusted operations library is the core component of the trusted operations framework. As depicted in Figure 3, the trusted operations library is integrated with the trusted application. It is a statically-linked library that provides a set of APIs to facilitate the implementation of trusted operations and execute data acquisition protocols. The trusted operations library is based on the GlobalPlatform TEE APIs [30]. The GlobalPlatform trusted application specifications require each TA to implement a number of functions called the TA interface. These functions are invoked by the TEE to notify events during the life-cycle of a TA [30]. The trusted operations library implements the TA interface and provides a single entry point as TO_Entry() to the TA. The TA can then register for the TA life-cycle events from within the TO_Entry function using event registration functions provided by the trusted operations library.

The trusted operations library provides APIs to register arbitrary operations with the framework. These operations are essentially callbacks that are called by the framework to perform the desired operations on the sensor data; while the framework takes care of performing seal/unseal or sign/verify operations before and after performing the operation on the sensor data.

Data acquisition is defined as an operation in the trusted operations library and can be registered as an operation that will return the sealed data from the sensor. In the case of the external sensor, the trusted operations library provides a predefined operation to initiate the external sensor session, while the corresponding data acquisition operation is registered with a special flag to denote external sensor data. In this case, the library performs the necessary verification steps for the external sensor data before it eventually seals and returns the data back to the REE application.

The trusted operations library also provides a special type of operation to merge more than one sensor reading together and create a unified seal for the merged data. Geotagging an image can be considered as an example of such merging; one example is the binding of air quality data from an external sensor with the GPS coordinates taken from the mobile phone’s on-board GPS. The framework relies on accurately time-stamping the sensor data during acquisition. These timestamps are compared before the merge operation, and the operation succeeds only if the two readings were taken within a predefined temporal proximity.

### 6.2. Trusted Data Lifecycle

**Figure 4:**
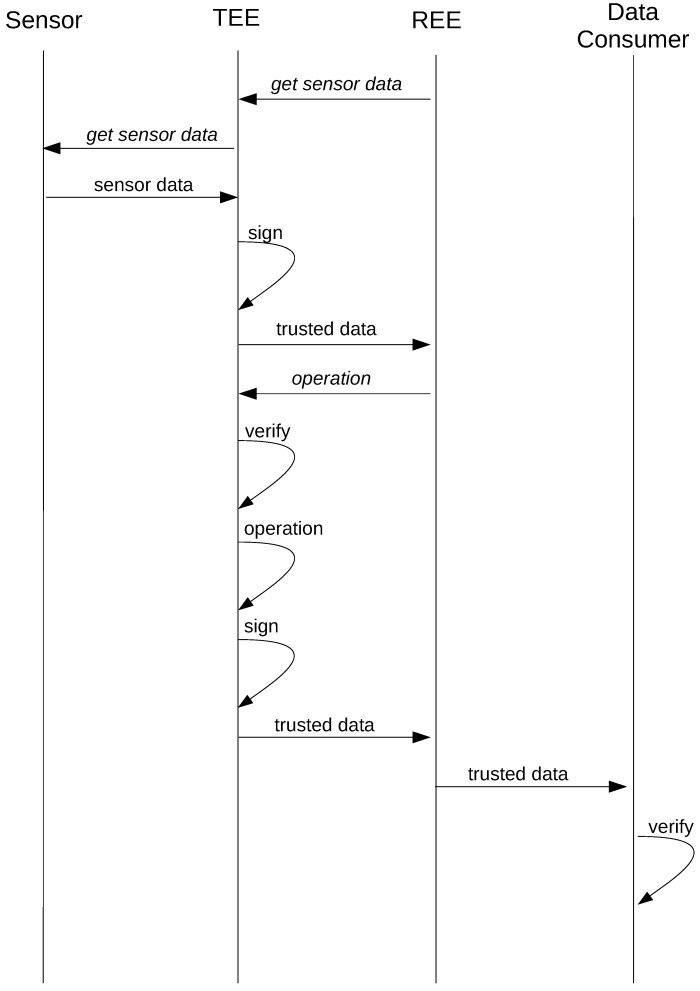
On-board sensor data life cycle.

Figure 4 illustrates the sequence of events that take place in the life cycle of the data squired from sensors. The application running in the REE initializes the data acquisition sequence by calling a function on the TA running in the TEE. Sensor data are obtained from on-board sensors simply by querying the sensor from the TA; however, reading data from an external sensor requires a much more elaborate sequence of steps, which will be explained in the following section. However, in both cases, the data are timestamped and sealed before sending them to the REE application.

Later, when the REE application needs to perform an operation on the data, it invokes the operation command and transmits the sealed data to the TA, which first unseals and verifies the data, then performs the designated operation, seals the resulting data and sends them back to the REE application. The REE application then transmits the sealed data to the data consumer, which typically is a cloud application, where again, the data are unsealed and are only accepted if they can be validated.

### 6.3. External Sensor Protocol

Sensors that are external to the mobile phone have added security considerations for providing a proof of freshness while simultaneously keeping the secrecy and integrity guarantees. In order to fulfill the freshness requirement, the external sensor needs to participate in a challenge/response sequence that should be completed within a predefined time limit. In order to ensure the freshness of the data, each external sensor data request is contained within its own time-bounded session with its unique session key, which is generated at the start of the session.

There are three main entities involved in the external sensor protocol. These entities are the TA, the sensor node and the verification server. All three entities have been initialized with their own pair of public/private keys. The public key of the verification server has been transported to both the TA and the sensor node, and the public keys of the sensor node and the TA have been transported to the verification server. This key distribution is later used for identity verification of the sensor node and the TA to prevent a man-in-the-middle attack during their communication. The identity of the sensor node is also verified via the verification server. This choice is made in favor of scalability as it is not possible to install public keys of all sensor nodes in all TAs.

The external sensor protocol works in two phases. The first phase is the key exchange and verification phase where a shared secret key is established between the TA running inside the TEE and the external sensor. The second phase is the data acquisition phase. It is triggered whenever the application requires to read sensor data from the external sensor. The key exchange phase requires network connectivity with the verification server where TA communicates with the verification server via the REE application. The communication between the TA and the verification server is secured using the previously-distributed public keys between the TA and the verification server. Furthermore, the TA does not have a direct communication channel with the external sensor, and all communication between the TA and the sensor node is relayed by the REE application.

**Figure 5:**
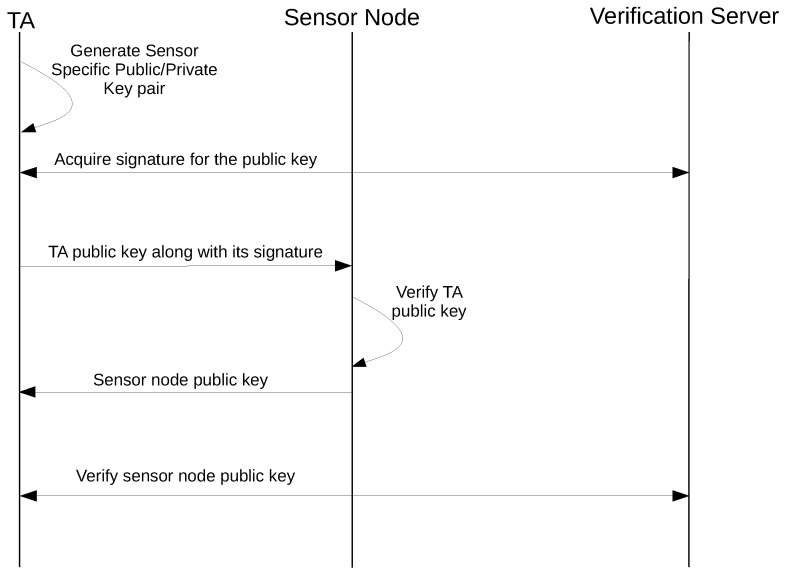
External sensor protocol: key establishment.

As shown in Figure 5, the key exchange phase consists of the following steps:

1. The TA generates a sensor-specific public/private key pair to be used for Diffie-Hellman key exchange.
2. The TA acquires a signature for its public key from the verification server.
3. The TA initiates the communication by sending its public key along with the signature provided by the verification server to the sensor node.
4. The sensor node verifies the signature for the TA public key using the (already installed) verification server public key. If the TA public key was verified, the sensor node generates a Diffie–Hellman shared secret using its own private key and the TA public key.
5. The sensor node sends its public key to the TA.
6. TA verifies the identity of the sensor node from the verification server and generates the Diffie–Hellman shared secret using its own private key and the sensor node public key.

**Figure 6:**
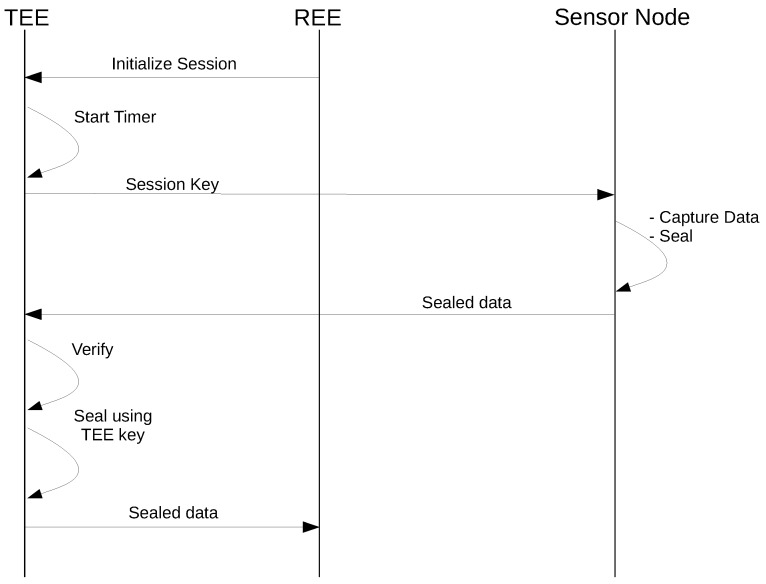
External sensor protocol: data acquisition

Once the sensor node is verified and a shared secret has been established between the TA and the sensor node, the application running in the REE initiates the data acquisition phase by calling the external sensor session initialize operation on the TA. As shown in Figure 6, the following sequence of steps takes place in the data acquisition phase.

1. Upon receiving a session initialization request from REE, the TA starts a timer and generates a unique random nonce called the TA nonce.
2. The TA sends the nonce to the external sensor in plain text.
3. The external sensor generates a new random nonce as the sensor nonce and then generates a session key using the two nonces and the previously-established shared secret.
4. The external sensor takes the sensor reading and seals the sensor data using the session key.
5. The sensor node transmits the sealed sensor data along with the sensor nonce.
6. The TA generates the session key using the same algorithm used by the sensor node, verifies the sealed data, checks for the session timeout, unseals the data and generates a TA-specific trust header for the data.
7. The TA then reseals the data using the TA private key.
8. The sealed data are then sent to the REE to be transmitted to the data consumer.

### 6.4. Design Goals

The trusted operations framework achieves the design goals listed as contributions in the Introduction section. The goal of providing secrecy and integrity of sensor data is achieved by the ‘seal’ and ‘unseal’ operations. Data confidentiality is ensured by applying the seal operation at the time of data capture inside the TEE, while the corresponding unseal operation can only be performed by a trusted party. This operation ensures that the REE application only has access to the encrypted sensor data. On the other hand, if only data integrity is required, another variant of the seal operation is used that attaches a signature generated using the TA private key to the sensor data. In this case, any tampering of the data is easily detectable as the signature can only be generated using the TA private key.

The trusted operations framework facilitates arbitrary operations on sensor data by providing an API to register operations developed by the TA developer. The TA needs to call TO_RegisterOperation() to register each operation to the framework. The trusted operations framework defines an operation as a TEE command, and the operations also take the same parameters as the TEE command. This allows the client application running in the REE to call the operation just as a TEE command; however, the trusted operations framework takes care of the seal/unseal operations and data verification while performing the said operation. After each successful operation, the trusted operations framework appends the operation code to the operations list to keep track of the chain of trust.

The trusted operations framework also supports a special type of operation that takes two sensor data parameters. In this case, the framework unseals/verifies both sensor data parameters and invokes the operation; the operation will bind the two sensor data according to its algorithm; and in the end, the framework will seal the resulting data.

In the case of external sensors, the framework takes care of two main security considerations. (1) The freshness of the data is achieved by using time-bounded sessions for data acquisition, and this ensures that the REE cannot present old sensor data in a new session. (2) External sensor data confidentiality is achieved by the seal/unseal operation similar to the internal sensors. The external sensor session management, key exchange and data transmission are defined by the external sensor protocol. This protocol is independent of the underlying transmission protocol. The evaluation section explains the adaptation of the protocol that uses BLE as the communication channel between the external sensor and the REE application. The trusted operations framework factors out the seal/unseal and verification operations, leaving only the core algorithm of the operations for the TA developer. This simplifies trusted operation development effort and, due to the reuse of code, reduces the footprint of the TA.

## 7. Implementation

We implemented the trusted operations framework proof of concept using OP-TEE running on Raspberry Pi 3. OP-TEE is open source, implements GlobalPlatform TEE and has been ported to many platforms suitable for academic and research purposes; This makes it an ideal choice for implementing the proof of concept trusted operations framework. The Raspberry Pi 3 has an ARM Cortex-A53 as its main processor, which implements Arm TrustZone [15]. The Raspberry Pi 3 processor provides ARM TrustZone exception states, which are sufficient for implementing a functional proof of concept TEE and trusted applications. However, Raspberry Pi 3 lacks secure boot, memory, peripherals and other security functions required by secure storage. Due to these limitations, a system implemented on Raspberry Pi 3 is only suitable for non-commercial applications. We used the TEE development setup created by Sequitur Labs [31] for the prototype implementation of the Trusted operations framework.

OP-TEE supports statically-linked, as well as dynamically-loadable Trusted Applications (TA). We implemented the proof of concept using dynamically-loadable ELF. The client-side of the framework utilizes the GlobalPlatform APIs directly. The only requirement from the client side is that the input and output data are preceded by a trust header consisting of the signature of the data as the first element, then a list of operations that have been performed on the data, followed by a timestamp indicating the time of capture of the data and lastly the sensor data.

### External Sensor

Our prototype implementation of the external sensor node is based on the BLE Nano board [32]. In its current state, the BLE Nano board does not fulfill the high security requirements required for being the root of trust. In order to make the BLE Nano board trustworthy, either it should support TEE of its own or have a TPM to ensure the protection of the secret keys. We simplify our prototype implementation by using the BLE Nano board in its original form and recommend that a professional implementation should consider more secure hardware as an external sensor node.

The BLE Nano board has a Nordic nRF51822 that is built around a 32-bit ARM Cortex-M0 with an embedded BLE transceiver. The firmware is programmed using Sloeber, the Arduino IDE for Eclipse [33]. BLE was used as the medium of communication between the sensor node and the mobile phone. Although BLE has built-in security features, we cannot rely on them as our threat model includes compromisable application software on the mobile phone. We used the analog pulse sensor from Electronics LLC [34] as the external data sensor in our implementation.

Similar to the internal sensors, the data acquisition and transmission of the data to the cloud data consumer are driven by the REE application. However, in the case of external sensors, the REE application also relays communication between the TA running in the TEE and the external sensor node. When the external sensor node is connected to the mobile phone for the first time, the TA fetches the public key of the external sensor and verifies the identity of the external sensor. The identity of the external sensor can be verified using a trusted third-party on the Internet; we do not go into the details of key distribution and identity verification in this paper and assume that after this step, the external sensor is verified. After verification, the TEE establishes a shared secret with the external sensor using Diffie–Hellman key exchange [35]. We used the key generation and exchange algorithms implemented in ArduinoLibs [36], which is based on elliptic curve modulo 2^255^ − 19 [37].

Later, when the REE initializes the data acquisition sequence, it first sends an initialize session command to the TA, which in turn starts an internal timer and generates a random session key; this session key is transmitted to the sensor node. The sensor node uses this session key as initialization vectors for encrypting the sensor data and the Message Authentication Code (MAC). The Nordic nRF51822 provides a hardware-based implementation of AES ECB. We utilize this hardware module to implement counter mode AES. The sensor data are divided into 20-byte encrypted packets consisting of 16 bytes of data and a four-byte MAC. The MAC is calculated by computing a SHA256 of the 16-byte data and encrypting first four bytes of the hash. The 20 bytes fit the maximum length of a BLE custom characteristic, which is used to transmit the secure data to the TA. After receiving the encrypted sensor data, the TA decrypts and verifies the data, performs the seal operation using the TA private key and sends the data to the REE application.

## 8. Evaluation

We evaluated our system based on the requirements that we captured from our scenario. The first subsection evaluates the security requirements for the system by following the flow of data from their origin at the sensor to the transmission of the data to the data consumer. At each step, we evaluate the security of the data. The second subsection evaluates the performance of the system and establishes the viability of performing the seal/unseal operations at each data acquisition. We assume that on-board sensors are used for a larger volume of data as compared to external sensors and evaluate the performance of these sensors accordingly.

### 8.1. Security

Sensor data can be captured using either on-board sensors on a mobile phone or external sensors attached to the mobile phone. We evaluate the security considerations for the two types of sensors separately.

When data are acquired form an on-board sensor, the root of trust is kept in the TEE. The sensor data are always acquired using a trusted operation call. This ensures that the data indeed originated from the desired sensor. After capturing the data, TA creates a trust header that includes the current timestamp, signature of the data and an empty list of operations that have been performed on the data. From here onward, the trust header is always attached to the data. This is essential for later verifying the authenticity of the data. There are two security elements in the trust header: (1) the timestamp and (2) the signature. The timestamp is generated using the TEE-controlled real-time clock, thus placing the root of trust in the TEE. The signature of the sensor data is generated using a hash of the sensor data combined with the timestamp. This hash is signed using the private key of the TA, again placing the root of trust in the TEE. The construction of the trust header ensures that the root of trust is always in the TEE and as a result ensures the security of the data at the time of data acquisition.

Secure acquisition of sensor data from external sensors is ensured by the external sensor protocol. The goal of the external sensor protocol is to create a trust header for the external sensor data. In order to analyze the protocol against known attacks, we review the external sensor protocol step by step and discuss security considerations on each step.

While analyzing the protocol, we assume that the initial key exchange between the protocol entities was performed in a secure manner. In Step 1 of the key exchange phase, a cryptographic key pair is generated within the TEE and stored in secure storage. The use of secure storage prevents keys from unauthorized access. In Step 2, the TA acquires a signature for its newly-generated sensor-specific public key from the verification server. Since the verification server and the TA already have each others’ public key, the communication between the TA and the verification server is authenticated using the public keys, thus eliminating any chance of a man-in-the-middle attack. In Step 4, the sensor node verifies the signature of the TA public key before proceeding with the generation of the shared secret key using the Diffie–Hellman key exchange algorithm. Here, the verification of the TA public key ensures that the public key is valid, which prevents an impersonation attack by an attacker pretending to be a TA. The shared secret generated at this stage is securely stored inside the external sensor node. In Step 6, the TA verifies the authenticity of the public key sent by the external sensor node from the verification server. This step ensures that the same public key sent by the external sensor node at Step 5 was received by the TA. This eliminates the possibility of a man-in-the-middle attack to trick the TA into accepting an incorrect key. Lastly, the TA generates the same shared secret as in Step 4 using its own private key and the sensor node public key as input to the Diffie–Hellman key exchange algorithm.

A unique random nonce is generated at the first step of the data acquisition phase. This nonce is used to mark the session uniquely and will prevent a replay attack that could resend the sensor data from a previous session. The TA also starts a timer to mark the start of the session. This timer is based on the secure TEE clock and is independent of the REE clock. In Step 2, the nonce is transmitted to the external sensor in plain text. In Step 3, the sensor node generates a sensor nonce and uses the TA nonce, the sensor nonce and the shared secret key to generate a session key. The sensor nonce is used to introduce randomness in the session key even when an attacker manages to replace the TA nonce with its own value. Without session key randomness, the sensor data could be inferred by analyzing more than one encrypted sensor data value that were encrypted using the same session key. In Steps 4 and 5, the sensor data are read by the sensor node, sealed using the session key and sent to the TA along with the sensor nonce. At this point, the TA has all the information to generate the session key and use this key to unseal the sensor data. If the sensor data arrived within the session timeout, the TA accepts the data and is now ready to create a trust header for the data. The session key ensures the fidelity and secrecy of the data, while the session timeout provides the proof of freshness for the data. These attributes ensure that the trust header created for the data can be trusted. After attaching the trust header, the external sensor data have the same trust guarantees as the data from internal sensors.

We also need to ensure security while performing operations on the data. In order to keep the root of trust in the TEE, all operations on the data are performed inside the TEE and only after verification of the trust header, and after performing the operation, the trust header is updated by inserting the operation code in the list of operations and updating the signature in the trust header. This makes the updated data as reliable as the original acquired data. Furthermore, since the framework keeps track of the operations performed on the data, a set of operations that could have changed the meaning of the data is also recorded by the system. This extra information is helpful in tracking the operations that can potentially change the meaning of the data.

The eventual destination for the sensor data is the data consumer. In most cases, this data consumer resides in a server on the cloud. Before accepting the data, the data consumer first verifies the data using its trust header and only accepts valid data.

Based on the above analysis, we can conclude that the trusted operations framework fulfills the security requirements for data originating from both on-board and external sensors by placing the root of trust in the TEE and the security of the external sensor.

### 8.2. Performance

We used our framework to implement trusted operations for camera input. The trusted operations are taken from a real-world scenario where an employee takes pictures of a designated area and sends them to a data server. The data server first verifies that the pictures were taken at the specified place, at the specified time, and the fidelity of the picture is verifiable after any transformations have been applied on the image such as image-resize or encoding in a compressed format.

We implemented trusted operations for images that used the trusted operations framework to show the effectiveness of the framework in segregating the trust establishment logic from the actual implementation of the operations. We ported the stb image library [38] and the TinyJPEG [39] for OP-TEE to implement the image operations. Since the OP-TEE does not provide a full set of libc and libm libraries, we also ported a subset of the newlib libm in order to provide math functions for the image processing library [40]. We simulated image capture from the camera by reading the image from a file. The capture image operation reads the image and passes it to the REE using the trusted operations framework. Before passing the image to the client, the framework attaches a signature with the image data along with the capture time and an empty operations list. This signature is later used to verify the fidelity of the data. The second operation is the resizing of the image, and the operation is implemented using the stb image processing library. Encode and decode operations are implemented using the TinyJPEG library. All operations are implemented independent of the fidelity checking. The trusted operations framework performs signature verification before the operation and signature generation after calling the actual operation.

To evaluate our system, we implemented a chain of three operations on image data. The input is simulated using an image fie containing raw RGB data. The first operation is an image capturing operation that reads the image data and returns them back to the client. Then, we performed a chain of operations that represents a natural flow of operations that a typical street surveillance system would perform. The system captures the image, resizes it to a smaller size and then encodes it to jpeg. This image along with its trust header will be sent to the data server, which will first verify its fidelity and then store it in its database.

**Figure 7:**
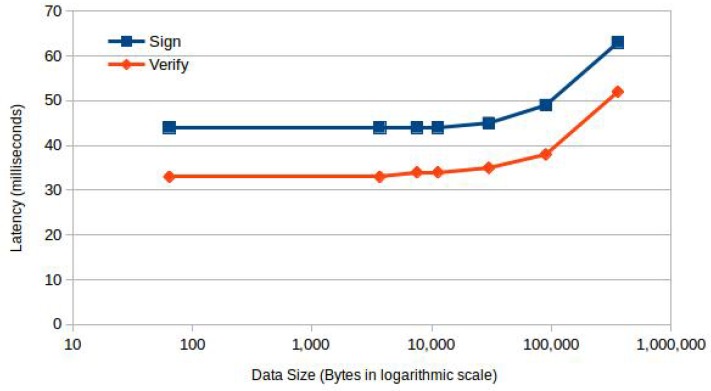
Sign/verify latency.

We computed the latency of calculating the hash, verifying and signing the data, using various sizes of data. As shown in Figure 7, the system incurs a constant overhead of 44 milliseconds for signing and 33 milliseconds for verification until 10 KB and increases linearly with the size of data afterwards. Evaluation results in [9] show that the average time of a sensor reading on an ARM TrustZone takes more than 80 milliseconds. The time delay incurred by sensor reading is greater than the time it takes to sign the sensor data; therefore, we conclude that fidelity validation is feasible at sensor data acquisition.

**Figure 8:**
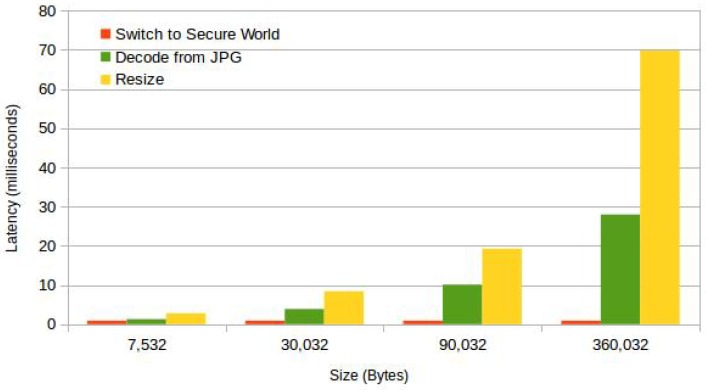
Operations vs. context switching.

Another interesting observation is that the time it takes in context switching from REE to TEE and back is negligible as compared to the time it takes to perform a typical image operation. This can be observed in Figure 8, where the latency of context switching and image operations is compared. In ARM TrustZone, both secure and normal worlds share the same processing resources; the context switching cost is the only overhead that is incurred by performing these operations in the TEE.

Sensor data acquisition and the operations performed on the sensor data are typically triggered as a result of user interaction. In this scenario, the overhead introduced by trusted operations is the combined cost of context switching, verification and signing. This overhead is less than 120 milliseconds for data of a size of 300 KB. We argue that this overhead is an acceptable delay in this scenario.

#### External Sensors

**Figure 9:**
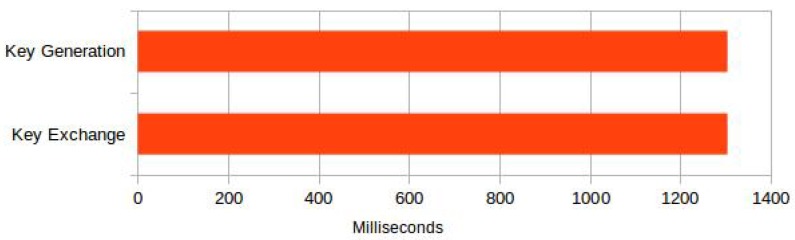
Shared secret establishment.

We evaluated the external sensors’ performance in two phases corresponding to key establishment and data transfer. In the first phase, the external sensor generates public/private key pair and establishes a shared secret with the TA in the mobile phone. The generation of secret keys and establishment of the shared key are the most computationally expansive operations in the protocol. However, secrete key establishment is independent of the sensor data exchange, and it is up to the user of the protocol to set the frequency by which this shared key is refreshed. As shown in Figure 9, it takes 1304 milliseconds to generate the Curve25519 public/private key pair and another 1304 milliseconds to establish the shared key using Diffie–Hellman key exchange.

**Figure 10:**
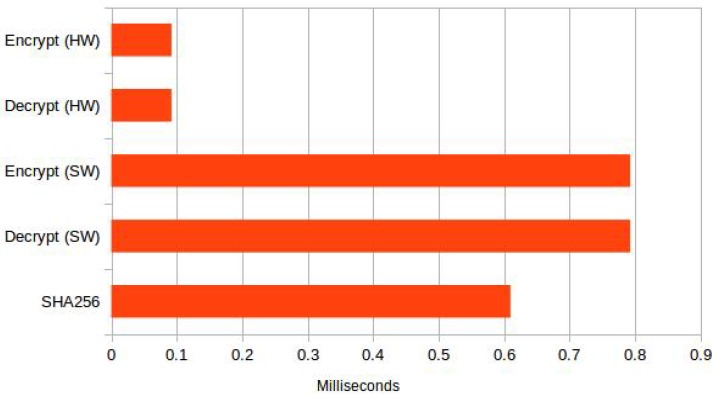
Seal/unseal external sensor data.

The second phase of the protocol starts with a data acquisition request from the mobile phone, which triggers data acquisition from the external sensor; the data are encrypted using AES counter mode encryption; a MAC is generated for the encrypted data; and the data along with the MAC are transmitted to the mobile phone. As shown in Figure 10, the encryption takes 92 microseconds for 16 bytes of data when hardware-based AES is used and 793 microseconds when software-based AES is use. Later, the MAC is calculated by calculating a SHA256 hash of the data and encrypting the hash. These operations cumulatively take 794 microseconds before the data are ready to be sent to the mobile phone. Even when we do not use the hardware-based implementation of AES, the total time to prepare the data is approximately 2196 microseconds, which is insignificant to the transmission delays. This low latency makes it viable to encrypt the data before transmitting them to the mobile phone.

Based on the performance evaluation of securing external sensor data, we conclude that using end-to-end security with the TA is indeed feasible, and the overhead caused by the added security is acceptable.

## 9. Future Work

This paper assumes that the key generation and public key transportation are performed in a controlled environment where an administrator manually triggers key generation and extracts the public key in order to register the device. Another approach is to use a third-party certification authority to provide digital certificates. In order to avoid manual key exchange or third-party certification authority, we plan to implement a public key distribution infrastructure based on distributed block-chain to create a network of trusted mobile devices. This key distribution mechanism will eliminate the need for a centralized key management entity and will enable installation of the trusted operations framework independent of a trusted administrative body.

## 10. Conclusions

This paper presents a framework that facilitates the implementation of trusted operations on sensor data. The framework abstracts out the implementation details of encrypt/decrypt and sign/verify sensor data and provides a clean way of implementing arbitrary operations that are to be performed on the sensor data, while the sensor data acquisition, signature generation and verification are handled by the framework itself. In addition to that, it provides a mechanism to capture sensor data from external sensors while providing the same security guarantees. This approach factors out the common code for performing security operations, thus reducing the footprint of the overall code and providing an opportunity to religiously test and verify the common code independent of the actual operations’ implementation. We provide a security analysis of the sensor data lifecycle, from both on-board, as well as external sensors and conclude that as long as the TEE on the mobile phone and the security of the external sensor are intact independently, it is possible to provide confidentiality and fidelity guarantees for the sensor and derived data. At the end, the paper presents the performance evaluation of the framework and an example of its usage for image operations implemented in OP-TEE running on a Raspberry Pi 3.

## Figures and Tables

**Table 1:** Comparison to related work.

Related Work	Integrity	Secrecy	Arbitrary Operations Supported	Root of Trust in TEE	Resilient to Compromised OS	External Hardware Required
[23]	Yes	No	No	No	Yes	Yes
[7,8]	Yes	Yes	Yes	No	Yes	Yes
[9]	Yes	Yes	No	Yes	Yes	No
[10]	Yes	No	No	No	No	No
[13]	Yes	No	No	No	No	No
[25]	Yes	Yes	Yes	No	Yes	Yes
Current Paper	Yes	Yes	Yes	Yes	Yes	No

## Abbreviations

The following abbreviations are used in this manuscript:

TEE	Trusted Execution Environment
REE	Rich Execution Environment
BLE	Bluetooth Low Energy
TA	Trusted Application
TPM	Trusted Platform Module
SGX	Software Guard Extensions
ELF	Executable and Linkable Format
AES	Advanced Encryption Standard
ECB	Electronic Codebook
